# Neuromusculoskeletal Modeling-Based Prostheses for Recovery After Spinal Cord Injury

**DOI:** 10.3389/fnbot.2019.00097

**Published:** 2019-12-02

**Authors:** Claudio Pizzolato, David J. Saxby, Dinesh Palipana, Laura E. Diamond, Rod S. Barrett, Yang D. Teng, David G. Lloyd

**Affiliations:** ^1^School of Allied Health Sciences, Griffith University, Gold Coast, QLD, Australia; ^2^Griffith Centre for Biomedical and Rehabilitation Engineering, Menzies Health Institute Queensland, Griffith University, Gold Coast, QLD, Australia; ^3^The Hopkins Centre, Menzies Health Institute Queensland, Griffith University, Gold Coast, QLD, Australia; ^4^Gold Coast Hospital and Health Service, Gold Coast, QLD, Australia; ^5^School of Medicine, Griffith University, Gold Coast, QLD, Australia; ^6^Department of Physical Medicine and Rehabilitation, Spaulding Rehabilitation Hospital, Harvard Medical School, Charlestown, MA, United States; ^7^Department of Neurosurgery, Brigham and Women’s Hospital, Harvard Medical School, Boston, MA, United States

**Keywords:** spinal cord injury, neuromusculoskeletal modeling, neural restoration, functional electrical stimulation, brain-computer interface, real-time, digital twin, rehabilitation robotics

## Abstract

Concurrent stimulation and reinforcement of motor and sensory pathways has been proposed as an effective approach to restoring function after developmental or acquired neurotrauma. This can be achieved by applying multimodal rehabilitation regimens, such as thought-controlled exoskeletons or epidural electrical stimulation to recover motor pattern generation in individuals with spinal cord injury (SCI). However, the human neuromusculoskeletal (NMS) system has often been oversimplified in designing rehabilitative and assistive devices. As a result, the neuromechanics of the muscles is seldom considered when modeling the relationship between electrical stimulation, mechanical assistance from exoskeletons, and final joint movement. A powerful way to enhance current neurorehabilitation is to develop the next generation prostheses incorporating personalized NMS models of patients. This strategy will enable an individual voluntary interfacing with multiple electromechanical rehabilitation devices targeting key afferent and efferent systems for functional improvement. This narrative review discusses how real-time NMS models can be integrated with finite element (FE) of musculoskeletal tissues and interface multiple assistive and robotic devices with individuals with SCI to promote neural restoration. In particular, the utility of NMS models for optimizing muscle stimulation patterns, tracking functional improvement, monitoring safety, and providing augmented feedback during exercise-based rehabilitation are discussed.

## Introduction

Spinal cord injury (SCI) partially or fully interrupts physiological connections between the brain, the spinal cord, and the muscles. Motor commands can still be generated but may not reach the muscles to produce movement. Similarly, sensory signals from below the injury site, such as signals indicating gravity, motion, touch, pain and/or temperature, cannot travel back to the spinal cord and brain for proprioceptive input to motor pattern generators, somatosensorimotor perception, and neocortical/conscious interpretation. This feedback loop must be reconnected if mobility, motor pattern generation, and sensation are to reoccur after SCI (Jackson and Zimmermann, 2012).

Recent advances in neural prosthetics and rehabilitation robotics have shown great promise for restoration of voluntary movement in individuals with SCI. These techniques have been shown to restore function in rats with a transected spinal cord (van den Brand et al., 2012). Direct electrical stimulation of the injured spinal cord with co-application of pharmacological agents, such as serotonergic receptor agonists, has also enabled individuals with SCI to regain some voluntary movement (Gerasimenko et al., 2015; Angeli et al., 2018; Gill et al., 2018; Sayenko et al., 2018). Researchers (Donati et al., 2016) recently combined motor-driven exoskeleton gait training and tactile feedback using simulated foot pressures (movement sensation) with advanced brain-computer interfaces (BCI) and virtual reality to restore the brain-muscle loop. Preliminary evidence suggests that concurrently engaging the central and peripheral nervous (e.g., propriospinal projection network, reticulospinal serotonergic neurotransmission) and muscular (e.g., proprioceptive receptors and neuromuscular junctions) systems may promote restoration of the central pattern generator of the spinal cord (i.e., neural restoration) (Ropper et al., 2017; Teng, 2019).

The abovementioned studies have raised expectations about the feasibility of designing non-invasive efficacious therapies for the SCI population, a prospect heretofore considered unattainable. However, application of these technologies often requires guesswork from clinicians or researchers to define the parameters associated with the amount of stimulation or support. For example, functional electrical stimulation (FES) requires users to specify frequency, duty cycle, current amplitude, and timing to achieve a predefined movement pattern. Parameters are usually predefined by the manufacturer, leaving the clinician to adapt the therapy to the patient based on trial-and-error approaches and clinical experience (Doucet et al., 2012). Similarly, motorized rehabilitation robotics require users to pre-select gait kinematics and/or kinetics, which are then used to drive the patient during rehabilitation. These sets of parameters need to be maintained within safe limits to prevent injury (He et al., 2017; Angeli et al., 2018). Otherwise, applying electrical stimulation or powered rehabilitation robotics can result in excessive tissue strains and consequent tissue failure given the atrophied musculoskeletal tissues and low bone density present in individuals with SCI (He et al., 2017). However, these approaches to assisted therapy are currently often not personalized to the patient, which could potentially result in poor patient engagement, and consequently, sub-optimal interaction-enhanced neural plasticity.

A neuromusculoskeletal (NMS) model is a physics-based functional representation of an individual’s NMS anatomy and physiology, which can be used to estimate the internal states of musculoskeletal tissues non-observable via instruments external to the body. A NMS model may account for individual-specific musculoskeletal capabilities, and be used to quantify the difference between voluntary muscle activation and the external assistance required to perform a specific task. NMS models may also monitor musculoskeletal tissue stress/strain to prevent injury, and quantify improvement following rehabilitation, such as increases in voluntary force. Using NMS models, existing rehabilitation methods could be further expanded and improved upon developing a personalized therapy that reduces clinician guesswork by automatically stimulating the patient’s muscles, adapting to the patient’s recovering muscle activation patterns, challenging the patient in recovery to maximize engagement, and maintaining the amount of external assistance within safe limits.

Here we intended to focus on how NMS models can be used to integrate different neuromechanical prostheses to maximize potency of neurorehabilitation following SCI. The review comprises an overview of currently available neuromechanical prostheses and describes how real-time NMS models can be integrated with assistive devices to improve rehabilitation outcomes. We concluded with a summary of current limitations of the presented approach and suggestions for future research directions.

PubMed was searched for articles published in English from January 1980 to October 2020. Search terms included “FES,” “BCI,” “neural prosthesis,” “exoskeleton,” “rehabilitation robotics,” “NMS modeling,” “finite element (FE) modeling,” and “digital twin.” Abstracts were reviewed, and papers with a focus on applications in SCI were further analyzed in detail.

## Neuromechanical Prostheses for Individuals With Spinal Cord Injury

A neuromechanical prosthesis can be defined as any device or combination of devices that support and/or replace any neural or mechanical function of an individual. In the context of SCI, neuromechanical prostheses to restore function include BCI, peripheral and spinal electrical stimulation, and rehabilitation robotics.

### Brain-Computer Interfaces

Brain-computer interface can capture the user’s intention to perform a movement, which can be used to control computer simulations and/or external electromechanical devices (Pfurtscheller et al., 2003a; Silvoni et al., 2011). The patient’s movement or force output is captured by the afferent pathways, which in turn affects the patient’s brain activity. Motor imagery, the act of imagining performing a movement without producing mechanical output, can modify the neuronal activity of the sensorimotor cortex, similar to what occurs when performing the real movement (Pfurtscheller and Neuper, 2001). Electroencephalogram (EEG) recordings acquired synchronously with motor imagery of tasks such as cycling or walking can be used to train a machine learning classifier to discriminate between different brain states (Lotte et al., 2018). Using this approach, BCI can then be used in real-time to classify different motor intentions and control assistive devices, such as rehabilitation robotics (Barsotti et al., 2015) and FES (Pfurtscheller et al., 2003b). BCI has also been used in combination with more sophisticated machine learning methods to predict kinematic of movement (Cheron et al., 2012) and control the gait of a virtual reality avatar in real-time (Luu et al., 2016). Practically, this means that EEG acquired by a BCI can be transformed into an output in the real world. Current evidence suggests rehabilitation using a BCI can induce neural plasticity and improve motor function in people with neurological conditions (Grosse-Wentrup et al., 2011).

### Peripheral and Spinal Electrical Stimulation

Electrical stimulation uses electrical current to stimulate the spine, peripheral nerves, or musculoneuronal junctions to artificially induce muscle contraction. Stimulation results in synchronous recruitment of motor neurons and force production at the level of muscle fibers. Force is then transmitted via tendons to the skeletal system producing final effector force and movement. Electrical stimulation of muscles, often referred to as FES, is non-invasive and can be performed transcutaneously via pairs of electrodes applied to each muscle or muscle group. FES during leg cycling is a popular rehabilitation modality (Ragnarsson, 2008), as the pedaling motion is a closed kinematic chain (i.e., constrained mechanical action) and accessible to people with tetraplegia. Even when implemented more than 20 years following SCI (Mohr et al., 1997), FES leg cycling continuously showed multiple clinical benefits, such as increased cardiorespiratory performance (Pollack et al., 1989) and endurance (Mohr et al., 1997), prevention of muscle atrophy (Baldi et al., 1998), and increased muscle mass (Mohr et al., 1997).

Epidural electrical stimulation of the spinal cord has been used to evoke rhythmic electromyograms (EMGs) from muscles of the lower limbs, resulting in individuals with complete SCI to independently walk again (Angeli et al., 2018; Gill et al., 2018). Less invasive transcutaneous electrical stimulation of the spinal cord also restored movement in the lower limbs of individuals with SCI, resulting in retention of some volitional movement control even in the absence of electrical stimulation (Gerasimenko et al., 2015). However, improper stimulation of the spinal cord may interfere with afferent neural pathways and disrupt proprioceptive information (Formento et al., 2018), which are proposed to be essential for restoration of neural function (Rushton, 2003). Moreover, there might be potential for adverse events when stimulation is applied to SCI patients using guesswork alone to define input parameters. Thus, technologies and methods for preventing excessive tissue loading in response to electrical stimulation are essential.

Treatments involving electrical stimulation necessitate a set of parameters to be defined, such as on/off timing of muscle stimulation, and stimulation frequency and amplitude. Clinically, these parameters are commonly set by the operator based on predefined values or via trial and error experiential approaches (Doucet et al., 2012). Automatic parameters selection can be achieved via closed-loop control strategies that automatically tune amplitude and/or frequency of stimulation to track predefined kinematics or kinetics targets (Hunt et al., 2004; Lynch and Popovic, 2008; Li et al., 2016). However, most control strategies presently available do not appropriately model the underlying NMS system of an individual, overly simplifying or ignoring the dynamics of muscle activation and contraction and their effects on joint movement (Sartori et al., 2016). These control strategies do not permit observation of the internal state of the musculoskeletal system, nor allow for planning optimal muscle coordination strategies when multiple degrees of freedom are involved. Moreover, even most recent approaches to controlling do not account for variations in an individual’s anatomy, physiology, or neuromuscular system, nor do they automatically adapt to a patient’s changing neural capabilities on any time scale. Collectively, these technologies have many limitations, that if addressed, could greatly enhance rehabilitation outcomes.

### Rehabilitation Robotics

Rehabilitation robotics involves any motorized electromechanical system, either wearable or stationary, that assists an individual to perform a target movement, such as exoskeletons (Jezernik et al., 2003) and motorized ergometers (Mekki et al., 2018). Robotic-assisted rehabilitation commonly involves securing the patient to the machine and the therapist defining what specific gait or cycling kinematics pattern the robot should provide. In most cases involving individuals with complete SCI, the patient is completely and passively guided by the robot and minimally engaged in the rehabilitation process, due to no need to deliver any kind of executive command from the brain. During this process, intact spinal loops and reflexes may be triggered, resulting in some amount of muscle contraction, force and movement generation, and may also contribute to maintaining overall musculoskeletal tissue health, but effectiveness of these therapies on walking function remains poor (Swinnen et al., 2010; Mehrholz et al., 2017). If these robotic assistive devices could be designed to maximally engage the individual’s motor imagery, this would be an improvement over current use of this technology.

### Combined Use of Multiple Assistive Devices

Multiple assistive devices have been combined to maximize functional outcomes (Mekki et al., 2018). In a study involving BCI-controlled FES of wrist, continuous and sustained motor imagery throughout FES resulted in greater cortical activity when compared to lack of motor imagery during FES (Reynolds et al., 2015), potentially suggesting strengthening of corticospinal pathways (Pfurtscheller and Lopes da Silva, 1999). A similar strategy was also used to successfully restore wrist motor function in post-stroke individuals, wherein FES was significantly more effective when controlled by a BCI (Biasiucci et al., 2018). Combining FES with rehabilitation robotics has been proposed to enhance devices’ performance (i.e., reduce the power of exoskeletons’ motors) (Ha et al., 2012, 2016) and to prolong the length of the rehabilitation session (del-Ama et al., 2014). Finally, BCI has also been combined with virtual reality and/or assistive robotic devices for rehabilitation of individuals with SCI (Donati et al., 2016).

The fast pace at which research involving neuromechanical protheses is growing (Marchal-Crespo and Reinkensmeyer, 2009) reveals an increasing need to integrate multiple assistive devices that can collaborate with the individual to promote adaptive and patient-centered therapy (Holanda et al., 2017). However, current approaches based on classic control theory or machine learning often oversimplify the complex dynamics of the human NMS system, omitting the mechanism underlying the causal relationship between observed input and output data (Sartori et al., 2016). Physics-based NMS models are an alternative approach that enables natural control of neuromechanical prostheses and permits assessing the internal state of an individual’s NMS system.

## Real-Time NMS Modeling to Integrate Assistive Devices

**FIGURE 1:**
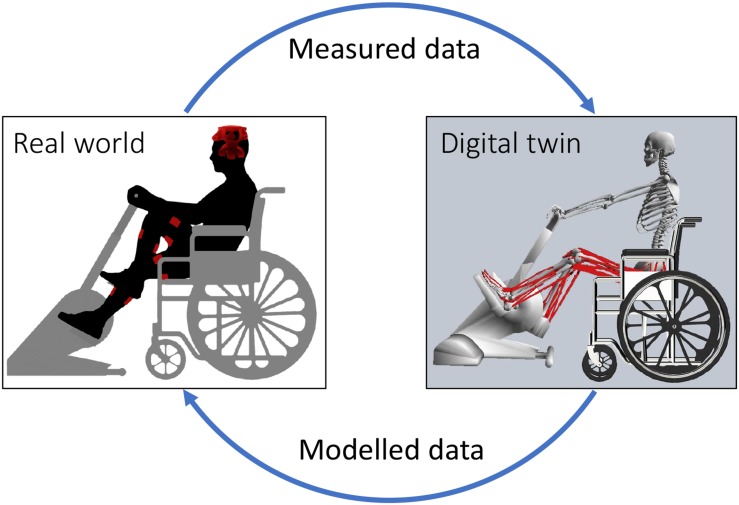
Schematic representation of the interaction between real world devices and digital twin. Data measured in the real world include physiological measurements from the individual, such as electroencephalograms (EEG) and electromyograms (EMG); and sensor data from assistive devices, such as force, torque, and position. EEG are used as input for machine learning methods to classify motor intention. Measured data and motor intention are then provided as input to digital twin of the patient and assistive devices. The digital twin implements a personalized NMS model of the individual that combines the input data to estimate optimal muscle activation patterns, localized musculoskeletal tissue stress and strains, and the amount of mechanical support that needs to be provided via rehabilitation robotics. Data modeled via the digital twin are then used to control assistive devices (e.g., electrical stimulation parameters and mechanical assistance) and provide augmented afferent feedback via visual and/or haptic monitors. This figure depicts a stationary ergometer and electrodes for functional electrical stimulation (FES), but the same concept can be applied to other types of rehabilitation robotics (e.g., exoskeletons) and electrical stimulation (e.g., epidural stimulation).

Electromyogram -informed NMS models used experimentally measured EMG to perform forward dynamics simulations of muscle dynamics and estimate musculoskeletal tissue states (Lloyd and Besier, 2003; Sartori et al., 2012; Pizzolato et al., 2015). Musculotendon units are modeled using a Hill-type structure, where an elastic tendon is in series with a contractile muscle fiber. Musculotendon units are connected to bones via insertion points and follow anatomically derived paths that wrap around bones, which are used to estimate musculotendon units’ lengths and moment arms. The skeletal system is defined by multiple segments (i.e., bones) connected by three-dimensional joints mobilized accordingly to their anatomical function (Seth et al., 2018). EMG-informed NMS models have been used to successfully estimate muscle forces, joint contact forces, and joint stiffness in the lower and upper limbs of individuals with a variety of neuromuscular conditions (Sartori et al., 2015; Konrath et al., 2017; Hall et al., 2018; Hoang et al., 2018, 2019; Lenton et al., 2018; Kian et al., 2019). NMS models are the optimal platform for integration of multiple assistive devices, enabling physics-based sensor fusion, where input and output quantities are mechanistically and causally related. This is equivalent to the modern concept of a digital twin (Glaessgen and Stargel, 2012; Boschert and Rosen, 2016), but here applied to a person and their assistive device(s) (Figure 1). Although not explicitly addressed as such, digital twins based on NMS models are becoming a core technology for human-machine interaction (Sreenivasa et al., 2019) and personalized rehabilitation (Sartori et al., 2016; Pizzolato et al., 2017a, 2019), promising exciting technological advancement in prosthetic limb control (Sartori et al., 2019).

Calibration of NMS model parameters is an identification procedure whereby an optimization algorithm finds the optimal set of parameters that minimize the error between experimentally observed and model-predicted quantities (e.g., joint movement, joint moments, joint powers, and EMG). Importantly, calibration ensures that non-observable quantities predicted by the model (e.g., muscle forces, joint contact forces) are physiologically plausible (Gerus et al., 2013; Hoang et al., 2018). Prediction of muscle force has been shown to be particularly sensitive to optimal fiber length, tendon slack length, and maximum isometric force (Scovil and Ronsky, 2006). Furthermore, parameters associated with muscle activation dynamics have also been shown to require calibration (Lloyd and Besier, 2003). Robotic devices (e.g., the motors of exoskeletons or cycling ergometers) with no stimulation can be used to acquire the joint moments generated by passive musculotendinous and ligamentous tissue (Yoon and Mansour, 1982), and to limit the range of motion of each joint within specific thresholds based on passive moments, both of which are parameters that can be directly input into a personalized musculoskeletal model of an individual with SCI. Similarly, electrically stimulating one muscle group at a time would isolate the effects of contractile properties and activation dynamics of specific muscles and their causal contribution to joint moments and movement. Thus, through performing a sequence of robotic movements and electrical stimulation of different muscle groups, it may be possible to create an automated protocol to identify critical neuromuscular parameters. Future research focused into these domains is required to establish robust protocols for model personalization in individual with SCI.

Following calibration, NMS models need to operate in real-time to appropriately control multiple assistive devices. This means that variables used for controlling or monitoring devices need to be calculated with minimum delay by the NMS model. Recently, large scale real-time EMG-informed NMS model involving multiple degrees of freedom and musculotendon units have been used to estimate muscle forces and joint contact forces for the full lower limbs using experimental EMGs and motion capture (Pizzolato et al., 2017b, c; Durandau et al., 2018). This was enabled by an optimized multi-threaded software architecture, where a publisher-subscriber software pattern was used to independently handle the multiple input and output devices minimizing idle times (Pizzolato et al., 2017b). Another advantage of this architecture is the ability to modify input or output devices independently from the underlying NMS model, in a plug-and-play fashion. This allows, for example, the operator to easily use wearable sensors, or any other device in place of stereophotogrammetry motion capture systems to acquire human motion in real-time. Output devices may include audio, visual and/or haptic monitors to provide augmented somatosensory information, or control commands for external devices, such as neuromechanical prostheses. The multithreaded software architecture used in recent real-time NMS modeling enables compete decoupling between input and output devices whereby the NMS model acts as super-controller and interpreter between the human and the machine (Ceseracciu et al., 2015). This decoupling allows NMS models to be adapted for a multitude of neurological conditions and neuromechanical prostheses. Finally, these EMG-informed NMS models have the potential to be applied to individuals with SCI to (i) generate optimal muscle stimulation, (ii) improvement tracking and safety monitoring, and (iii) augment afferent feedback.

### Generating Optimal Muscle Stimulation

Successful rehabilitation for SCI patients will involve generating an appropriate set of muscle activation patterns that account for an individual’s capabilities. From optimal activation patterns required to generate the desired kinematic or kinetic task, it is then possible to calculate the required amplitude and frequency of stimulation for FES. However, individuals with SCI have different levels of neuromuscular dysfunction, with varying ability to produce voluntary muscle activations (Kirshblum et al., 2011). To further promote neural restoration, it is necessary to engage the patient such that they actively participate in the rehabilitation process. Examples of this approach involve EMG-gated FES, where muscle stimulation is provided only when concurrent voluntary contraction from the participant is present (Burridge and Ladouceur, 2001). This approach has been shown to produce better outcomes than standard non-EMG-gated FES (Dutta et al., 2009), though muscle stimulation occurs only if the patient is able to produce sufficiently large voluntary EMG, for which some patients are unable due to the severity of their injury. Furthermore, inter-individual anatomical differences in musculotendon lengths, moment arms, as well as differences in seating position and overall movement kinematics will result in different joint forces and moments for a given FES profile (Schutte et al., 1993). NMS models can appropriately account for these inter-individual differences, providing causal relationships between muscle stimulation and produced force.

A NMS model can be combined with a model of an electromechanical device to calculate an optimal set of muscle activation patterns required to perform a rehabilitation task. In a closed kinematic chain, such as in cycling, the hip, knee, and ankle joint angles are determined as a function of the crank and pedal angles. Thus, joint moments can be easily calculated when the desired values for average power output and cadence are provided (Farahani et al., 2014). NMS models are readily used in conjunction with mathematical optimization (e.g., static optimization) to estimate the optimal set of muscle activation pattern required to perform a predetermined movement. The difference between the voluntary muscle activations of the patient and the target muscle activations from the NMS model provides an objective basis for calculation of the FES compensation level (Yeom and Chang, 2010). Using NMS models, it is also possible to estimate the contribution of voluntary activation from each muscle of the patient to the final joint moments. Consequently, the ratio between the support provided to the patient via electrical stimulation of muscles and the support provided via mechanical assistance can be modulated, which introduces the possibility of creating advanced control strategies that reward patient engagement and voluntary muscle contractions.

A NMS model can be used as layer between a BCI and multiple assistive devices, transforming high level efferent neural commands into appropriate signals to control electrical stimulation and rehabilitation robotics. EEG acquired while an individual with SCI attempts to perform coordinated movement, such as cycling or walking, can be classified in real-time using machine learning approaches, and used to control a NMS model. A primitive but currently feasible solution would involve triggering the NMS model to perform a predetermined trajectory (e.g., cycling). Future approaches may explore BCI to extract basic spinal primitives that can be mapped to individual muscle to enable intuitive control of NMS models (Ubeda et al., 2018). This solution may be more advantageous compared to direct control of joint angles (Fitzsimmons et al., 2009) as it better reflects the current understanding of how the mammalian central nervous system organizes large groups of synergistic muscles during complex movement.

### Tracking of Improvements and Safety Monitoring

Globally, health-care system models are being redesigned to move from volume-based healthcare to value-based healthcare, which is organized around meeting a set of patient needs over the full care cycle (Porter et al., 2013). Although SCI patients consistently rate recovery of paralyzed limb function as their main priority (Anderson, 2004), clinical assessment alone is subjective and unable to quantify meaningful changes that precede major clinical and functional breakthroughs. Objective measurement of musculoskeletal states via NMS models can monitor a patient’s state and identify required adjustments in therapy, such as muscle-specific functional improvements. These measurements also have an important strategic objective: to define trajectories of rehabilitation to better inform guidelines for best-clinical practice so therapists and service providers can tailor patient support during the most critical stages of care.

Critical variables that reveal patient improvement can be tracked during NMS model-based therapies, which include the amount of robotic assistance required to perform the task and calibrated neuromuscular parameters assessed at each session. The mechanisms underlying any reduction in robotic assistance, such as increased volitional muscle activation or force production, may also be tracked. Such improvements may be below the detectable thresholds of standard clinical assessments, especially if the improvement is not sufficiently large to generate observable changes in movement. Similarly, musculoskeletal parameters calibrated at each session, such as maximum isometric force, could provide longitudinal measures of neuromuscular adaptation.

Individuals with SCI experience tremendous tissue atrophy (Giangregorio and McCartney, 2006), often losing up to 55% of muscle cross-sectional after 6 months from initial injury (Castro et al., 1999). If, through therapy, inappropriate loads are applied to these weakened tissues, tissue failure may occur (Angeli et al., 2018). Powered exoskeletons and rehabilitation robotics that have been recently approved by the United States Food and Drug Administration (He et al., 2017) as medical devices are expected to be increasingly marked; however, appropriate risk mitigation strategies to prevent injury are lacking (He et al., 2017). Additionally, incorrectly applied magnitude and timing of electrical stimulation to muscles can trigger pain that adversely affects rehabilitation-induced functional recovery and quality of life (Turtle et al., 2018). These risks may partially be mitigated using NMS modeling approaches.

**FIGURE 2:**
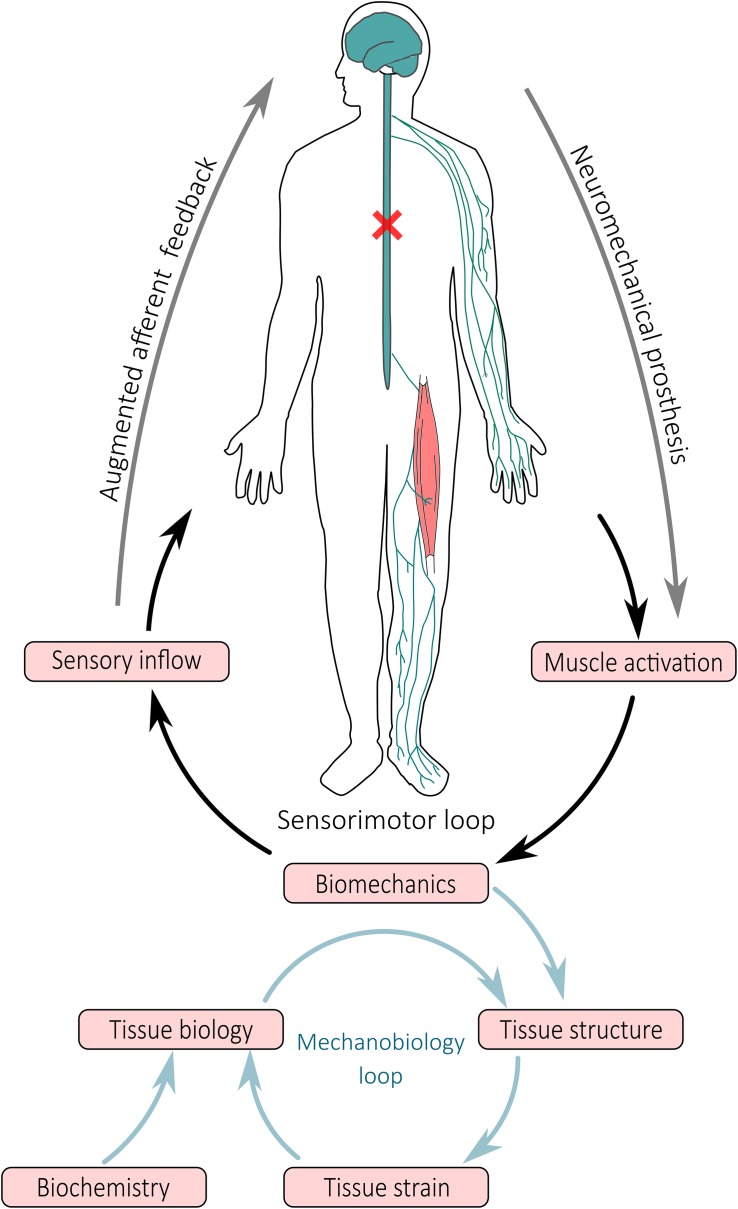
Schematic representation of closed-loop neuromechanical prostheses and their effect on movement and tissue adaptation. Neuromechanical prostheses interface with the central and peripheral nervous system bypass the spinal cord injury to modulate sensorimotor spinal loops, wherein activation of muscles and mobilization of joints result in limb movement and generation of sensory inflow via the somatosensory apparatus. Afferent signals synthetized by a digital twin of the person are redirected via alternative pathways to higher brain areas. At tissue level, the biomechanics (i.e., movement and muscle contraction) result in forces that are applied to the structure of tissues, generating a local mechanical environment (i.e., tissue strain) that modulates tissue biology and consequent tissue structural adaptation.

The FE method is a computational method that can be used to predict tissue damage or rupture by modeling the internal mechanics of tissue (i.e., localized stress and strain), as demonstrated by *ex vivo* studies (Shim et al., 2014, 2018). The geometry of FE models can be personalized to the individual via medical imaging (e.g., magnetic resonance imaging, x-ray computed tomography, or ultrasound) (Devaprakash et al., 2019), while material properties are typically applied from literature data or estimated experimentally (Hansen et al., 2017; Shim et al., 2019). Individual-specific boundary conditions calculated from NMS models (i.e., model pose and applied external forces) are supplied to FE models to estimate the internal stresses and strains of selected musculoskeletal tissues. However, FE analysis is computationally intensive and consequently, cannot be executed in real-time. Surrogate models of FE models have been developed for muscles (Fernandez et al., 2018), tendons (Shim et al., 2018), and bones (Ziaeipoor et al., 2019), enabling rapid evaluation of stress and strain patterns. Given a FE model of a tissue of interest, surrogate models are created in an offline process whereby a FE model is first solved for a complete set of physiologically plausible boundary conditions (e.g., known joint ranges of motion and applied muscle forces). Stress and strain data from all solutions are then used in conjunction with machine learning methods, such as partial least square regression, to create a surrogate model able to replicate the complete FE model with minimal computational complexity in real-time (Ziaeipoor et al., 2019). Surrogate FE models (Fernandez et al., 2018; Ziaeipoor et al., 2019) are currently being combined with real-time EMG-informed NMS models (Pizzolato et al., 2017c) to provide instantaneously estimates of tissue stresses and strains, as described in Pizzolato et al. (2017a, 2019). This is an exciting development, as it is now possible to combine NMS and FE models that are personalized to the individual to generate muscle activation patterns that ensure musculoskeletal tissues are loaded within safe limits. Furthermore, it is now feasible to objectively assess the effects of a rehabilitation exercise on the tissue-level signals that regulate the mechanobiology of musculoskeletal tissues (i.e., stress and strain), which could be coupled with tissue mechanobiology models (Mehdizadeh et al., 2017) to predict long term tissue adaptation following quantifiable mechanical stimulation (Pizzolato et al., 2017a; Figure 2).

### Augmented Afferent Feedback

In people with SCI, afferent signaling from somatosensory receptors below the level of injury is hindered, abnormal or absent. To maximize likelihood of neural restoration, afferent signals need to be redirected to intact somatosensory areas for neocortical and conscious interpretation (Jackson and Zimmermann, 2012). It remains unclear what somatosensory signals are most critical to augment technologically, the minimum amount of afferent feedback required, or the preferred delivery modality. It has, however, been proposed that efferent and afferent stimulation need to be consistent and synchronized to enable plastic remodeling of the central nervous system (Rushton, 2003; Jackson and Zimmermann, 2012). Consistent with what has been shown in motor learning and biofeedback studies (Kannape and Blanke, 2013; Sigrist et al., 2013), the delay in delivering technology-augmented afferent feedback must be minimized in order for patients to associate their own movement with the augmented afferent feedback. Using this principle, a recent study employing robotically assisted and virtual reality gait retraining measured plantar pressure transformed into haptic feedback delivered via pads applied to the shoulder region to provide movement sensation (Donati et al., 2016). Further, visual feedback via virtual reality has been used to display patient’s avatar limbs during BCI training (Donati et al., 2016), and augmented reality has been used to provide somatosensory feedback non-intrusively via peripheral vision (Clemente et al., 2017).

Neuromusculoskeletal models can be used to augment afferent somatosensory feedback and synthesize mechanoreceptors signals that are not externally observable (Pizzolato et al., 2017a,c). However, synthesized signals need to be integrated into a meaningful single or multi-modal feedback that can be easily interpreted by the patient (Sigrist et al., 2013). Peripheral nerve stimulation has been used to induce sensory feedback in amputees (Dhillon et al., 2004), and the same technique could be applied to redirect NMS model-synthesized afferent signals to intact sensorimotor areas for natural integration to mechanosensing feedback. Cortical interfaces are an alternative but invasive solution that could also be used to relieve burden from the visual system (Tomlinson and Miller, 2016). Cortical interfaces use an electrode microarray that is directly implanted into the sensorimotor cortex to provide electrical stimulation that mimics the natural cortex activity (Tomlinson and Miller, 2016), partially restoring proprioceptive function. Although still in its infancy, cortical interfaces have shown promise in animal studies, and could, in the future, be combined with multiple assistive devices to maximize neural restoration in individual with SCI.

## Limitations and Future Directions

A variety of assistive technologies are currently available to aid the rehabilitation of individuals with SCI. However, significant improvements and recovery of motor function have been predominantly shown when these devices were combined rather than used in isolation. In this review we have proposed integrating these different technologies via computational NMS models. These models can be considered as a digital twin of the patient and their devices acting as an interpreter between human and machine, continuously monitoring internal tissue state, and tracking longitudinal changes throughout the rehabilitation journey. Nonetheless, several challenges will need to be addressed to achieve this goal, which will require the combined effort from multidisciplinary research engagements.

Current approaches to BCI based on motor imagery are not sufficiently robust and require retraining neural decoders at the beginning of each rehabilitation session (Lotte et al., 2018). Furthermore, only a few movements can be classified via this approach, limiting the use of BCI to simple motor tasks (Bamdad et al., 2015). Part of the problem resides in the poor signal to noise ratio, spatial resolution, and inter-session variability of the EEG signals acquired at the scalp. More robust classification methods able to adapt to the user are currently being explored by the BCI research community, but an optimal classification method is yet to be established (Lotte et al., 2018). Recently developed minimally invasive implantable BCI have been able to acquire EEG for extended time periods with greater signal quality than superficial EEG (Oxley et al., 2016). In the future, this technology may enable superior classification of motor intention and seamless integration of humans with assistive devices.

Our proposed strategy involves using NMS models personalized to the individual; however, current personalization methods involve time consuming semi-automatic processing of medical imaging data (Valente et al., 2017). Machine learning methods to automatically segment tissue from medical imaging [e.g., neural networks (Zhou et al., 2018)] and to generate personalized models from population databases [e.g., statistical shape modeling (Suwarganda et al., 2019)] are emerging as promising technologies to personalize anatomy and function of NMS models. However, further efforts will be required to simplify the creation of these models via seamless processing pipelines (Zhang et al., 2014) in order to enable their routine clinical use.

The same NMS modeling-based approach described here for individuals with SCI can be applied for neurorehabilitation of other types of acquired neurological impairments, such as traumatic brain injury and stroke. NMS model-based neuromechanical prostheses are currently possible and within reach, but these assistive technologies will need to be co-designed with clinicians, care providers, and patients to develop devices that are fit for purpose and aligned with the expectations of the final users. If accepted by the clinical community, NMS modeling approaches to neurorehabilitation have the potential to reduce current clinical guesswork by automatically adapt to the individualized needs of each patient, enabling minimally supervised rehabilitation sessions, and reducing costs of care. Clearly, efficacy of NMS modeling-based neuromechanical prostheses will first need to be addressed in clinical trials to understand the effect of exercise dosage, afferent feedback modality, and pharmacological agents on rehabilitation outcomes. Overall, personalized NMS models have the potential to improve current assistive technologies and potentiate neural recovery after SCI.

## Author Contributions

CP contributed to conceptualize, draft, critically revise, and approve the final version of the manuscript. DS, DP, LD, RB, YT, and DL contributed to conceptualize, critically revise, and approve the final version of the manuscript.

## Conflict of Interest

CP, DS, DP, LD, and DL have applied for a patent regarding the use of neuromusculoskeletal-modeling-based controllers with robotic devices and brain-computer interfaces to synthesize efferent and afferent signals to restore function after spinal cord injury described in this review. The remaining authors declare that the research was conducted in the absence of any commercial or financial relationships that could be construed as a potential conflict of interest.
